# Telemedicine infectious diseases consultations and clinical outcomes: a systematic review and meta-analysis protocol

**DOI:** 10.1186/s13643-019-1056-y

**Published:** 2019-06-07

**Authors:** Jason P. Burnham, Stephanie A. Fritz, Lauren H. Yaeger, Graham A. Colditz

**Affiliations:** 1 Division of Infectious Diseases, Washington University School of Medicine, 4523 Clayton Avenue, Campus Box 8051, St. Louis, MO 63110 USA; 2 Department of Pediatrics, Washington University, St. Louis, MO USA; 3 Bernard Becker Medical Library, Washington University, St. Louis, MO USA; 4 Division of Public Health Sciences, Washington University School of Medicine, St Louis, MO USA

**Keywords:** Systematic review, Telemedicine, Infectious diseases, Consultation, Mortality, Telehealth, Cost, Antimicrobial

## Abstract

**Background:**

Telemedicine use is increasing in many specialties, but its impact on clinical outcomes in infectious diseases has not been systematically studied and reviewed. The proposed systematic review will evaluate the current evidence regarding the effect of telemedicine infectious diseases consultation on a range of clinical outcomes, including mortality, hospital readmission, antimicrobial use, and cost.

**Method/design:**

Standard systematic review methodology will be used, with searches of Ovid MEDLINE 1946-, https://embase.com/ 1947-, Scopus 1823-, Cochrane Database of Systematic Reviews (CDSR), Cochrane Central Register of Controlled Trials (CENTRAL), and https://clinicaltrials.gov/ 1997-. There will be no restriction on language or year of publication. The primary outcome will be 30-day all-cause mortality and secondary outcomes will include readmission within 30 days after discharge from an initial hospitalization with an infection, patient compliance/adherence, patient satisfaction, cost or cost effectiveness, length of hospital stay, antimicrobial use, and antimicrobial stewardship. Bias will be assessed using standard Cochrane methodologies. Data will be grouped by outcome and narratively synthesized. Meta-analysis will be performed for outcomes with clinical or methodological homogeneity. The systematic review and meta-analysis will be registered through PROSPERO. Pre-planned subgroup analyses will be detailed.

**Discussion:**

A number of studies have documented the feasibility of telemedicine for infectious diseases, but a synthesis of clinical outcomes data with telemedicine infectious diseases consultation has not been performed. This systematic review will analyze many clinical outcomes of telemedicine infectious diseases consultation. The findings of this study will add to established literature about feasibility of telemedicine consultation by synthesizing the evidence for clinical effectiveness.

**Systematic review registration:**

PROSPERO CRD42018105225

**Electronic supplementary material:**

The online version of this article (10.1186/s13643-019-1056-y) contains supplementary material, which is available to authorized users.

## Background

According to recent estimates, infectious diseases may be the third leading cause of death in the USA [1]. Underserved or economically disadvantaged areas often do not have access to infectious diseases (ID) physicians (up to 45% of US hospitals) to help treat these infections [2]. Consultation with ID physicians has been shown to significantly reduce mortality for a range of infections [3, 4]. Providing access to ID expertise in underserved areas could substantially reduce mortality and improve other clinical outcomes. The field of ID has difficulty recruiting new physicians, with 51% of fellowship programs not filling in 2015 [5, 6]. With this shortage of ID physicians, it is not feasible for remote locations to employ a dedicated ID physician. Telemedicine could efficiently and cost-effectively expand ID expertise to underserved areas. Telemedicine reduces mortality in progressive and intensive care units and in very low-birth weight infants [7–9], but there has been no synthesis of evidence for the use of telemedicine for infectious diseases. Our systematic review and meta-analysis will address this deficiency by summarizing the evidence for clinical effectiveness of telemedicine for infectious diseases.

## Methods/design

### Aim

The aim is to assess the effectiveness of telemedicine for all patients with infectious diseases for a range of clinical outcomes (enumerated below) as compared to either (1) no infectious diseases consultation or (2) other modalities of infectious disease consultation (e.g., in-person). The aim will be achieved by conducting a systematic review of studies in which telemedicine is utilized to study any of the following clinical outcomes: 30-day all-cause mortality, readmission within 30 days after discharge from an initial hospitalization with an infection, patient compliance/adherence, patient satisfaction, cost or cost effectiveness, length of hospital stay, antimicrobial use, and antimicrobial stewardship.

Standard systematic review methodology will be used, with searches of Ovid MEDLINE 1946-, Embase.com 1947-, Scopus 1823-, Cochrane Database of Systematic Reviews (CDSR), Cochrane Central Register of Controlled Trials (CENTRAL), and Clinicaltrials.gov 1997-. The protocol has been registered with the international prospective register of systematic reviews (PROSPERO), in accordance with PRISMA-P guidelines [10].

The search strategy includes terms for infection, telehealth, and clinical outcomes and has been submitted to PROSPERO in advance of study initiation. The full search strategy will be released upon completion of the study. Articles included in the final systematic review and meta-analysis will be reviewed for other references that were missed by the search strategy. Systematic reviews will also be used to determine if all relevant primary studies have been identified.

There will be no language or year of publication restrictions. Translation of non-English language articles will be undertaken, either in part or in full, as required. Conference abstracts will be excluded if sufficient outcome and bias data cannot be extracted from the abstract. Duplicate records will be purged. REDCap will be used by the authors for data entry.

### Selection criteria

In order for studies to be included in the final analysis, they must meet all of the following inclusion criteria:

- Populations/participants must receive telemedicine consultation for an infectious disease or be a control arm of a study of telemedicine consultation for an infectious disease
- Report one or more of the following clinical outcomes: 30-day mortality after an infection, readmission within 30 days after discharge from the initial hospitalization with an infection, patient compliance/adherence, patient satisfaction, cost or cost effectiveness, length of stay, antimicrobial use, and antimicrobial stewardship

Studies will be excluded if any of the following conditions are met:

- Case report only
- Conference abstracts in which insufficient detail is provided to ascertain outcomes or determine the possibility of bias
- Do not have one of outcomes of interest measured
- If a study involves consultation for multiple conditions, the study will be excluded if outcomes specifically for infectious diseases cannot be separated from outcomes for other conditions
- No control group
- Non-interventional studies including, but not limited to, reviews, protocols, editorials, letters to the editor, and viewpoints

### Definitions

Telemedicine will be defined as remote clinical services administered using a technological medium. This includes face-to-face video chat (physician-to-physician or physician-to-patient), voice chat after review of electronic health records, or electronic health record documentation after remote chart review without direct voice or video contact with physician or patient.

Where the data are available, we will categorize hospital readmissions as being related or unrelated to infection. For the outcome of cost effectiveness, we are specifically referring to a comparison of cost between telemedicine and usual care (i.e., control) groups for an episode of infection. Antibiotic stewardship will be quantified as either antibiotic costs or antibiotic appropriateness, as judged by the authors of the individual studies.

### Selection process

Titles and abstracts will be reviewed by JPB, with studies excluded that obviously do not meet inclusion criteria or meet any exclusion criteria. After the initial title and abstract review, in a blinded fashion, JPB and GAC will review the relevant full-text articles to determine their relevance to the research question. Any disputes on potential study inclusion will be settled by a third reviewer (SAF—also blinded). Details of the reasons for exclusion at the full-text stage will be recorded and reported. Study selection will be documented using a PRISMA flow diagram.

### Data extraction

In a blinded fashion, two authors (JPB and GAC) will independently extract data from the included articles. Discrepancies will be adjudicated by a third reviewer (SAF). Data will be entered into a data extraction form through REDCap. Data to be extracted include study quality, clinical or system-level outcome tracked, percent change or proportion experiencing each clinical outcome, and numbers of patients in all intervention arms as well as percent receiving ID consults, age group, consultant specialty, type of telemedicine, study location, whether infection was confirmed by laboratory results, and type and risk of bias.

### Quality assessment

Risk of bias will be reviewed by two reviewers (JPB and GAC) in a blinded fashion. Any disputes on potential bias will be settled by a third reviewer (SAF—also blinded). Bias determination will be guided by the applicable portions of the Cochrane Consumers & Communication Review Group Study Quality Guide or Newcastle-Ottawa scale [11, 12].

### Analysis

We plan to analyze primary and secondary outcomes with a quantitative synthesis for all participants, but also plan to analyze by age (children < 18 versus adult ≥ 18 years), by whether the telemedicine consultant is infectious diseases trained or not, by infection type, by type of telehealth/telemedicine intervention (e.g., face-to-face, asynchronous), by study location in the USA versus non-US study location, by number of ID consultations (i.e., days), and by culture or laboratory-confirmed infection versus presumed infection. Qualitative synthesis will be performed if there are too few studies.

For each outcome, if clinical and methodological heterogeneity are sufficient, meta-analysis will be performed using either fixed or random effect models [13]. If more than 10 studies are included in the meta-analysis for a particular outcome, we will investigate for publication bias using standard methodologies [14].

We will also perform a descriptive review of the contextual use of telemedicine, including infection type and screening criteria for telemedicine enrollment.

### Reporting

The findings of this review will be published in accordance with PRISMA-P guidelines [10] (see Additional file 1).

## Discussion

Infectious diseases are a major source of morbidity and mortality in the USA and abroad. The field of ID has difficulty recruiting new physicians, with 51% of fellowship programs not filling in 2015 [5, 6]. As such, strategies are urgently needed to increase access to ID expertise to improve patient outcomes. Our study will systematically review and analyze the evidence for the use of telemedicine for infectious diseases to help determine whether it is an appropriate medium for dissemination of ID expertise. If our study shows benefit for telemedicine as a means of managing infectious diseases, it will be a prime research target for dissemination and implementation research.

## Additional file

Additional file 1: PRISMA-P 2015 Checklist. (DOCX 29 kb)
